# Real-world efficacy and safety of two doses of cabazitaxel (20 or 25 mg/m^2^) in patients with castration-resistant prostate cancer: results of a Japanese post-marketing surveillance study

**DOI:** 10.1186/s12885-020-07131-6

**Published:** 2020-07-13

**Authors:** Hideyasu Matsuyama, Nobuaki Matsubara, Hirotaka Kazama, Takeshi Seto, Shoko Tsukube, Kazuhiro Suzuki

**Affiliations:** 1 Department of Urology, Graduate School of Medicine, Yamaguchi University, Yamaguchi, Japan; 2 Department of Breast and Medical Oncology, National Cancer Center Hospital East, Kashiwa, Japan; 3 Sanofi Genzyme Oncology Medical, Sanofi K.K., Tokyo, Japan; 4 Medical Affairs, Sanofi K.K., Tokyo, Japan; 5 Department of Urology, Gunma University Graduate School of Medicine, Maebashi, Japan

**Keywords:** Cabazitaxel, Castration-resistant prostate cancer, Post-marketing surveillance, Japan

## Abstract

**Background:**

The recommended starting dose of cabazitaxel for castration-resistant prostate cancer (CRPC) is 25 mg/m^2^ in Japan and Europe. Although lower doses are established alternatives based on randomized controlled trials, the safety and efficacy of 25 and 20 mg/m^2^ in real-world settings are not well established. Therefore, we investigated the safety and efficacy of cabazitaxel at the recommended starting dose or a lower dose (20 mg/m^2^) in real-world clinical practice.

**Methods:**

We compared the safety and efficacy of cabazitaxel between patients who received cabazitaxel at starting doses of 25 and 20 mg/m^2^ (C25 and C20, respectively) in a Japanese post-marketing surveillance study of 662 patients with docetaxel-refractory CRPC. Safety was assessed in terms of adverse drug reactions (ADRs). Prostate-specific antigen (PSA) response rate, overall survival (OS), and time-to-treatment failure (TTF) were compared between the C25 and C20 groups in unmatched patients and after applying propensity score matching.

**Results:**

The C20 and C25 groups comprised 190 and 159 patients without matching and 112 patients per group after matching. In unmatched patients, any-grade (C25 vs C20: 89.3% vs 78.4%, Fisher’s *p* < 0.01) and grade ≥ 3 (81.1% vs 61.1%) ADRs were more frequent in the C25 group. Neutropenia (any grade: 61.6% vs 54.2%; grade ≥ 3: 55.3% vs 42.6%) and febrile neutropenia (grade ≥ 3: 30.2% vs 14.7%) were more frequent in the C25 group. In matched patients, the PSA response rate (reduction in PSA ≥30% from a baseline ≥5 ng/mL) was 26.4 and 32.0% in the C20 and C25 groups, respectively, median OS was 291 days (95% CI 230–not reached) versus not reached (hazard ratio 0.73, 95% CI 0.50–1.08), and TTF favored C25 (hazard ratio 0.75, 95% CI 0.57–0.99).

**Conclusions:**

Clinicians should consider the patient’s risk of clinically significant ADRs and prophylactic granulocyte colony stimulating factor when selecting the starting dose of cabazitaxel for CRPC. Some patients at high risk of ADRs or unfit patients may benefit from a lower starting dose of 20 mg/m^2^, whereas fit patients may be candidates for a starting dose of 25 mg/m^2^.

**Trial registration:**

Not applicable.

## Background

Prostate cancer (PC) is a relatively common type of cancer with a generally high survival rate. In Japan, for example, the age-standardized prevalence of PC was 30.4 per 100,000 person-years and the mortality rate was 5.0 per 100,000 person-years [1]. The age-standardized 5-year survival rate in Japan also increased from 85.9% in 2000–04 to 93.0% in 2010–14 [2], perhaps resulting from improved treatments. However, many patients develop castration-resistant PC (CRPC) despite androgen deprivation therapy, and require additional therapy [3].

In recent years, several treatment options have been introduced for CRPC, including the novel androgen receptor-axis-targeted agents (enzalutamide and abiraterone), the radionuclide radium-223, and the new taxane, cabazitaxel [4, 5], which have since been incorporated into the treatment for CRPC in daily practice [6, 7].

Cabazitaxel is a second-generation taxane that was approved in the US in 2010 and Europe in 2011 following the international TROPIC study [8]. It was subsequently approved in Japan in 2014 based on pharmacokinetic studies confirming its pharmacokinetics and safety in Japanese patients were consistent with global findings [9, 10]. It has a safety profile consistent with that of first-generation taxanes [11–13]. The recommended initial dose of cabazitaxel is 25 mg/m^2^ in Europe and Japan. However, some studies suggested that a lower dose of 20 mg/m^2^ might be appropriate in consideration of safety [13, 14], and in some cases, adverse events (AEs) can be managed by patient monitoring and reducing the dose of cabazitaxel [15].

The appropriateness of 20 mg/m^2^ as a starting dose (C20), as compared with 25 mg/m^2^ (C25), was evaluated in PROSELICA, an international, randomized controlled trial [16]. The study showed that C20 was associated with a lower rate of treatment-emergent AEs of any grade and grade ≥ 3 AEs, with non-inferiority of overall survival (OS) (13.4 vs 14.5 months; hazard ratio [HR] 1.024), while the prostate-specific antigen (PSA) response rate (29.5% vs 42.9%, *p* < 0.001) and time to PSA progression (5.7 vs 6.8 months; HR 1.195) both favored C25. Nevertheless, because of the highly selected cohort enrolled in that randomized trial, the results may not reflect the outcomes in real-world settings involving heterogeneous populations.

Following its approval in Japan in 2014, a post-marketing surveillance study (PMS) of cabazitaxel was implemented to monitor its safety and tolerability for the treatment of CRPC in real-world clinical practice [17]. All treatment decisions were at the attending physician’s discretion, in consideration of treatment guidelines for CRPC and the package insert for cabazitaxel, which recommended an initial dose of 25 mg/m^2^. However, it was found that the starting dose was < 25 mg/m^2^ in 461 patients (69.8%) and the dose per cycle was < 25 mg/m^2^ in 542 patients (82.1%). The aims of the present report are to compare the safety and efficacy between two doses of cabazitaxel, namely 25 mg/m^2^ as the recommended dose (C25 group) and 20 mg/m^2^ as a low dose (C20 group), in real-world conditions, and to evaluate the appropriate starting dose in Japanese patients.

## Methods

### Study design, patients, and treatments

As previously described [17], the design of this PMS was reviewed by the Japanese Pharmaceutical and Medical Devices Agency and it was conducted in compliance with the Ministerial Ordinance on Good Post-marketing Study Practice for Drugs in Japan. This ordinance waives the need for ethical approval at participating institutions for studies of this type. Informed consent was not obtained, in accordance with these regulations and because data were collected using anonymous case-report forms, which could not be linked to the patient.

Briefly, this all-patient PMS was designed to enroll all patients with docetaxel-refractory CRPC who were scheduled to start cabazitaxel from September 2014 onwards [17]. Registration of patients was expected to continue for 4 years or up to 500 patients had been registered, whichever came first. The investigators provided data for patients who started and discontinued treatment or who completed treatment < 1 year after the start of cabazitaxel treatment and in patients who continued treatment for ≥1 year from the start of cabazitaxel treatment. As this was a non-interventional PMS, all treatment decisions were at the attending clinician’s discretion according to local treatment recommendations and the prescribing information. This included the dose and schedule of cabazitaxel, prophylaxis, and concomitant therapy. Prophylactic granulocyte colony stimulating factor (G-CSF) could be used to help prevent febrile neutropenia, and was recommended following an amendment to the package insert for cabazitaxel made in December 2014.

The participating clinicians completed case-report forms before their patients started taking cabazitaxel and during each treatment cycle. The information captured using these forms is described in more detail in our previous article [17], and included demographic and disease characteristics, previous and concomitant PC treatments, and PSA levels. Case-report forms were completed in each treatment cycle to document the use of cabazitaxel, prednisolone, premedications, concomitant drugs, and prophylactic G-CSF, as well as PSA levels, and information about any AEs/adverse drug reactions (ADRs) that occurred. The grade and type of AEs or ADRs were evaluated using Common Terminology Criteria for Adverse Events version 4.0. We also evaluated priority survey items as described in our previous report [17].

Survival was assessed up to 1 year after starting treatment. AEs/ADRs were recorded in the safety observation period, defined as the shortest period from starting cabazitaxel administration to 30 days after the last administration of cabazitaxel or to the first administration of cabazitaxel after completing 1 year of treatment. Efficacy was assessed in terms of the PSA response rate, OS, and time-to-treatment failure (TTF).

Patients were selected for matching based on the initial doses of cabazitaxel (20 mg/m^2^ [C20] and 25 mg/m^2^ [C25]) without dose escalation above the initial dose and the following on-label criteria: prior history of docetaxel treatment and concomitant administration of prednisolone.

### Statistical analyses

Patient baseline characteristics and safety outcomes were summarized descriptively, with data reported as the median (range), mean (standard deviation), and number (percent) of patients as appropriate. The rate of any-grade ADRs was compared between the C20 and C25 groups using Fisher’s test; rates of ADRs of individual grades and rates of individual ADRs were analyzed descriptively, without statistical testing to avoid multiplicity of analyses.

In order to control for possible differences in patient and disease characteristics that might confound the comparisons of efficacy, we performed propensity score matching (PSM) and multivariable analyses with logistic regression for PSA and the Cox regression model for OS and TTF by including the following 17 variables as covariates: age, body surface area, duration of disease, Gleason score, T classification, N classification, M classification, ECOG PS, PSA, medical history, complications, curative intent focal therapy, palliative radiation therapy, switch from docetaxel, number of docetaxel treatment cycles, reason for discontinuation of docetaxel, and previous treatment with enzalutamide or abiraterone. After including all of these variables, 1:1 matching (without replacement) was performed using the propensity scores with the nearest neighbor method and caliper width set to 0.2 standard deviations [18]. We examined the balance in baseline and matched variables by calculating the standardized difference scores. A standardized difference score of < 0.20 indicates acceptable balancing.

PSA response rates were calculated as the number (percent) of patients with decreased PSA level of 30% or more from a baseline level of ≥5 ng/mL. Odds ratios and 95% CIs were estimated by logistic regression. OS and TTF were calculated as the time from the start of treatment to death or discontinuation of cabazitaxel, respectively. The Kaplan–Meier method was used to determine the median OS and TTF with 95% CIs. In subgroup analyses, the HR with 95% CI was calculated for C20 vs C25 using the Cox regression model.

All tests were performed at a significance level of 5%. SAS 9.2 or 9.4 (SAS Institute, Cary, NC, USA) was used for all data analyses.

## Results

### Patients

**Fig. 1:**
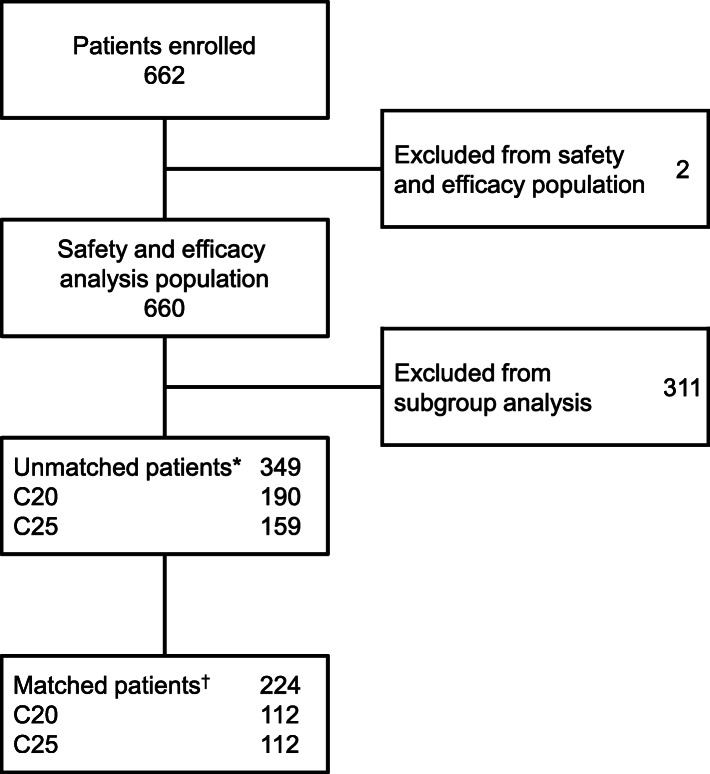
Patient disposition. *Patients who satisfied the following criteria: treatment with docetaxel before cabazitaxel; administration of prednisolone with cabazitaxel; and the cabazitaxel dose was not escalated above the initial dose during the treatment period. ^†^Patients were matched using propensity scores on 17 factors: age, body surface area, duration of disease, Gleason score, T classification, N classification, M classification, ECOG PS, PSA, medical history, complications, curative intent focal therapy, palliative radiation therapy, switch from docetaxel, number of docetaxel treatment cycles, reason for discontinuation of docetaxel, and previous treatment with enzalutamide or abiraterone. C20, 20 mg/m^2^ cabazitaxel; C25, 25 mg/m^2^ cabazitaxel

A total of 660 patients were registered in the PMS and received at least one dose of cabazitaxel. Of these, 349 patients satisfied the criteria for inclusion in the present analyses; 190 and 159 patients received cabazitaxel at initial doses of 20 mg/m^2^ (C20 group) and 25 mg/m^2^ (C25 group). After applying PSM, the C20 and C25 groups each comprised 112 patients (Fig. 1). Before matching, there were some apparent differences between the C20 and C25 groups for several baseline characteristics, including ECOG PS, PSA at baseline, medical history, complications, switching from docetaxel, and palliative radiation therapy (Table 1). Following PSM, the standardized difference scores for these variables were < 0.10, indicating better matching than a value of < 0.20 taken to represent acceptable matching.

**Table 1:** Patient characteristics

		All patients (*n* = 660)	Unmatched (*n* = 349)			Matched (*n* = 224)^a^		
			C20	C25	SD score	C20	C25	SD score
N		660	190	159	–	112	112	–
Age, years		70.0 (43–91)	71.0 (51–89)	69.0 (44–83)	0.271	69.5 (51–89)	70.0 (51–83)	0.051
Body surface area, m^2^		1.65 (1.26–2.20)	1.63 (1.26–2.02)	1.65 (1.30–2.20)	0.046	1.66 (1.26–2.02)	1.66 (1.30–2.20)	0.016
Duration of disease, years		4.16 (0.5–19.8)	4.71 (1.0–17.5)	3.85 (0.8–19.8)	0.059	4.10 (1.0–17.5)	3.71 (0.8–19.8)	0.069
Gleason score	2–7	104 (15.8)	28 (14.7)	27 (17.0)	0.064	14 (12.5)	15 (13.4)	0.025
	8–10	516 (78.2)	149 (78.4)	125 (78.6)		98 (87.5)	97 (86.6)	
TNM stage	T1 + T2	113 (17.1)	37 (19.5)	30 (18.9)	0.013	20 (17.9)	22 (19.6)	0.045
	T3 + T4 + TX	538 (81.5)	149 (78.4)	128 (80.5)		92 (82.1)	90 (80.4)	
	N0	297 (45.0)	94 (49.5)	72 (45.3)	0.068	50 (44.6)	53 (47.3)	0.054
	N1 + NX	359 (54.4)	93 (49.0)	86 (54.1)		62 (55.4)	59 (52.7)	
	M0	190 (28.8)	58 (30.5)	45 (28.3)	0.054	32 (28.6)	30 (26.8)	0.040
	M1 + MX	466 (70.6)	131 (69.0)	112 (70.4)		80 (71.4)	82 (73.2)	
ECOG PS	0	412 (62.4)	109 (57.4)	100 (62.9)	0.126	68 (60.7)	67 (59.8)	0.018
	≥1	247 (37.4)	81 (42.6)	58 (36.5)		44 (39.3)	45 (40.2)	
PSA (at baseline), ng/mL		164.9 (0.0–16,697.2)	146.1 (0.0–10,027.1)	173.2 (0.3–9892.3)	0.110	120.8 (0.0–4286.0)	187.6 (0.3–9892.3)	0.068
Previous medical history		202 (30.61)	65 (34.2)	44 (27.7)	0.159	36 (32.1)	37 (33.0)	0.019
Complications		275 (41.67)	82 (43.2)	61 (38.4)	0.146	42 (37.5)	43 (38.4)	0.018
**Previous treatments**								
Radical local excision		212 (32.12)	62 (32.6)	49 (30.8)	0.061	31 (27.7)	33 (29.5)	0.039
Switch from docetaxel		114 (17.27)	38 (20.0)	26 (16.4)	0.172	21 (18.8)	21 (18.8)	0.000
New-generation AR inhibitors^b^								
0 or 1 agents		340 (51.5)	96 (50.5)	89 (56.0)	0.010	60 (53.6)	59 (52.7)	0.018
2 agents		319 (48.3)	94 (49.5)	70 (44.0)		52 (46.4)	53 (47.3)	
Docetaxel chemotherapy		630 (95.5)	186 (97.9)	154 (96.9)	0.070	112 (100)	112 (100)	0.086
Docetaxel treatment cycles		9.0 (1–143)	9.5 (1–47)	9.0 (1–52)		10.0 (1–47)	8.0 (1–38)	
Reason for discontinuing docetaxel	PD	534 (80.9)	161 (84.7)	130 (81.8)	0.085	93 (83.0)	92 (82.1)	0.024
	AE/other	108 (16.4)	29 (15.3)	26 (16.4)		19 (17.0)	20 (17.9)	
Palliative radiation therapy		197 (29.8)	69 (36.3)	47 (29.6)	0.182	36 (32.1)	34 (30.4)	0.038

Values are reported as the median (range) or n (%)

*C20* 20 mg/m^2^ cabazitaxel, *C25* 25 mg/m^2^ cabazitaxel, *SDscore*, standardized difference score, *TNM* Tumor-node-metastasis, *ECOG PS* Eastern Cooperative Oncology Group performance status, *PSA* Prostate-specific antigen, *AR* Androgen receptor, *PD* Progressive disease, *AE* Adverse event

^a^Patients were matched using propensity scores on 17 factors: age, body surface area, duration of disease, Gleason score, T classification, N classification, M classification, ECOG PS, PSA, medical history, complications, curative intent focal therapy, palliative radiation therapy, switch from docetaxel, number of docetaxel treatment cycles, reason for discontinuation of docetaxel, and previous treatment with enzalutamide or abiraterone

^b^Enzalutamide or abiraterone acetate

### Cabazitaxel exposure

Cabazitaxel exposure was assessed in terms of the cumulative dose, actual dose intensity, and relative dose intensity (RDI) (Table 2). In matched patients, the median (range) cumulative dose was 80 (20–300) and 100 (25–400) mg/m^2^ in the C20 and C25 groups, respectively, with median RDIs of 65.9% (28.4–80.5%) and 77.0% (26.3–101.0%), respectively. The mean number of cycles and mean duration of treatment were both numerically greater in the C25 group.

**Table 2:** Cabazitaxel exposure

	All patients	Unmatched patients		Matched patients	
		C20	C25	C20	C25
N	660	190	159	112	112
**Number of cycles**					
Mean ± SD	5.5 ± 4.1	4.9 ± 3.7	5.8 ± 4.2	5.1 ± 3.7	5.9 ± 4.3
Median (range)	4.0 (1–18)	4.0 (1–15)	4.0 (1–16)	4.0 (1–15)	4.0 (1–16)
**Duration of treatment (days)**					
Mean ± SD	152.9 ± 119.2	134.9 ± 103.3	159.3 ± 121.6	138.5 ± 104.2	166.0 ± 126.5
Median (range)	106 (21–385)	103 (21–384)	110 (21–385)	112 (21–384)	110 (21–385)
**Cumulative dose (mg/m**^**2**^**)**					
Mean ± SD	114.4 ± 88.7	96.7 ± 71.2	134.5 ± 99.2	100.6 ± 72.7	136.4 ± 101.5
Median (range)	84.4 (10–445)	69.2 (20–300)	100.0 (25–400)	80 (20–300)	100 (25–400)
**ADI (mg/m**^**2**^**/week)**					
Mean ± SD	5.7 ± 1.4	5.4 ± 1.0	6.5 ± 1.5	5.4 ± 0.9	6.4 ± 1.5
Median (range)	5.6 (1.5–8.4)	5.4 (2.4–6.9)	6.6 (2.2–8.4)	5.5 (2.4–6.7)	6.4 (2.2–8.4)
**RDI(%)**					
Mean ± SD	68.0 ± 16.4	64.6 ± 11.6	78.3 ± 17.7	65.1 ± 11.3	76.6 ± 18.4
Median (range)	67.2 (17.8–101.0)	64.6 (28.4–82.6)	79.2 (26.3–101.0)	65.9 (28.4–80.5)	77.0 (26.3–101.0)

*C20* 20 mg/m^2^ cabazitaxel, *C25* 25 mg/m^2^ cabazitaxel, *SD* Standard deviation, *ADI* Actual dose intensity, *RDI* Relative dose intensity (planned dose intensity = 8.33 mg/m^2^/week)

### Safety

Rates of ADRs in the overall patient population and in the unmatched C20 and C25 groups are shown in Table 3, which includes a listing of all grade ≥ 3 ADRs that occurred in ≥2 patients each. The rates of ADRs in the overall patient population are discussed in more detail elsewhere [17]. Briefly, among 660 patients, 511 patients (77.4%) experienced 1113 ADRs and 409 patients (62.0%) experienced grade ≥ 3 ADRs, the most common being neutropenia, neutrophil count decreased, febrile neutropenia, anemia, and diarrhea.

**Table 3:** Adverse drug reactions by preferred term

	All patients				Unmatched patients							
	All grades		Grade ≥ 3		C20				C25			
					All grades		Grade ≥ 3		All grades		Grade ≥ 3	
N	660				190				159			
Patients with any ADR	511 (77.4)		409 (62.0)		149 (78.4)		116 (61.1)		142 (89.3)*		129 (81.1)	
Number of ADRs	1113		644		317		177		323		211	
ADRs (Grade ≥ 3 in ≥2 patients), n (%)												
Pneumonia	6	(0.9)	5	(0.8)	2	(1.1)	2	(1.1)	2	(1.3)	1	(0.6)
Pyelonephritis	3	(0.5)	3	(0.5)	0		0		1	(0.6)	1	(0.6)
Sepsis	4	(0.6)	4	(0.6)	2	(1.1)	2	(1.1)	1	(0.6)	1	(0.6)
Septic shock	3	(0.5)	3	(0.5)	0		0		1	(0.6)	1	(0.6)
Anemia^a^	99	(15.0)	58	(8.8)	22	(11.6)	12	(6.3)	25	(15.7)	15	(9.4)
Febrile neutropenia	119	(18.0)	113	(17.1)	28	(14.7)	28	(14.7)	50	(31.5)	48	(30.2)
Leukopenia^b^	74	(11.2)	48	(7.3)	23	(12.1)	13	(6.8)	18	(11.3)	15	(9.4)
Neutropenia^c^	324	(49.1)	263	(39.8)	103	(54.2)	81	(42.6)	98	(61.6)	88	(55.3)
Thrombocytopenia^d^	77	(11.7)	36	(5.5)	29	(15.3)	11	(5.8)	16	(10.1)	7	(4.4)
Bone marrow failure	2	(0.3)	2	(0.3)	0		0		1	(0.6)	1	(0.6)
Decreased appetite	49	(7.4)	11	(1.7)	17	(9.0)	3	(1.6)	10	(6.3)	2	(1.3)
Neuropathy peripheral	10	(1.5)	2	(0.3)	4	(2.1)	2	(1.1)	2	(1.3)	0	
Interstitial lung disease	8	(1.2)	7	(1.1)	1	(0.5)	0		4	(2.5)	4	(2.5)
Pneumonitis	2	(0.3)	2	(0.3)	0		0		0		0	
Diarrhea	66	(10.0)	21	(3.2)	21	(11.1)	5	(2.6)	16	(10.1)	6	(3.8)
Nausea	22	(3.3)	4	(0.6)	9	(4.7)	4	(2.1)	5	(3.1)	0	
Vomiting	11	(1.7)	3	(0.5)	1	(0.5)	0		3	(1.9)	2	(1.3)
Liver disorder	3	(0.5)	3	(0.5)	0		0		1	(0.6)	1	(0.6)
Malaise	40	(6.1)	3	(0.5)	8	(4.2)	0		9	(5.7)	0	
Pyrexia	22	(3.3)	4	(0.6)	7	(3.7)	1	(0.5)	4	(2.5)	1	(0.6)

*p < 0.01 vs C20 (Fisher’s test)

*C20* 20 mg/m^2^ cabazitaxel, *C25* 25 mg/m^2^ cabazitaxel, *ADR* Adverse drug reaction

^a^Anemia and hemoglobin decreased

^b^Leukopenia and white blood cell count decreased

^c^Neutropenia and neutrophil count decreased

^d^Thrombocytopenia and platelet count decreased

When we compared the rates of ADRs between the unmatched C20 and C25 groups, we found that the rate of any-grade ADRs was greater in the C25 group (89.3% vs 78.4%, *p* < 0.01). The rate of grade ≥ 3 ADRs was 81.1% in the C25 group and 61.1% in the C20 group. The rates of several ADRs, including neutropenia (any grade: 61.6% vs 54.2%; grade ≥ 3: 55.3% vs 42.6%) and febrile neutropenia (any grade: 31.5% vs 14.7%; grade ≥ 3: 30.2% vs 14.7%) were numerically greater in the C25 group. The rates of other ADRs, including diarrhea, thrombocytopenia, leukopenia, and anemia, were generally consistent between the C20 and C25 groups.

### Efficacy

#### PSA response rates

PSA response was defined as a reduction in PSA of ≥30% in patients with a baseline level of ≥5 ng/mL. Overall, 177 patients in the C20 group and 146 patients in the C25 group were eligible for the analysis of PSA response. As indicated in Table 4, there were no significant differences in the PSA response rates between the two groups, regardless of the matched analysis; in matched patients, the PSA response rate was 26.4 and 32.0% in the C20 and C25 groups, respectively.

**Table 4:** PSA response rate, overall survival, and time-to-treatment failure

Outcome	C20		C25		Statistic	
	N	Response rate, n (%)	N	Response rate, n (%)	OR (95% CI)	*p*
PSA response	601	169 (28.1)^b^				
Unadjusted	177	49 (27.7)	146	46 (31.5)	1.20 (0.74–1.94)	0.453
Multivariable^a^	135	38 (28.2)	120	36 (30.0)	1.18 (0.66–2.13)	0.572
PSM	106	28 (26.4)	103	33 (32.0)	1.31 (0.72–2.39)	0.372
**Outcome**	**N**	**Median (95% CI)**	**N**	**Median (95% CI)**	**HR (95% CI)**	***p***
OS (days)	656	319 (293–361)^b^				
Unadjusted	188	287 (240–326)	159	NR	0.71 (0.53–0.97)	0.030
Multivariable^a^	141	287 (234–327)	129	NR	0.69 (0.48–0.99)	0.047
PSM	110	291 (230–NR)	112	NR	0.73 (0.50–1.08)	0.119
TTF (days)	660	116 (108–135)^b^				
Unadjusted	190	113 (94–137)	159	120 (104–157)	0.78 (0.62–0.97)	0.026
Multivariable^a^	143	115 (92–138)	129	115 (99–157)	0.71 (0.54–0.93)	0.014
PSM	112	122 (90–148)	112	120 (109–158)	0.75 (0.57–0.99)	0.046

*C20* 20 mg/m^2^ cabazitaxel, *C25* 25 mg/m^2^ cabazitaxel, *PSA* Prostate-specific antigen, *OR* Odds ratio, *CI* Confidence interval, *PSM* Propensity score matching, *HR* Hazard ratio, *OS* Overall survival, *NR* Not reached, *TTF* Time-to-treatment failure

^a^Covariates were: age, body surface area, duration of disease, Gleason score, TNM, performance status, PSA, medical history, complications, curative intent focal therapy, palliative radiation therapy, and previous treatment (docetaxel, enzalutamide or abiraterone acetate)

^b^In all available patients

#### OS

**Fig. 2:**
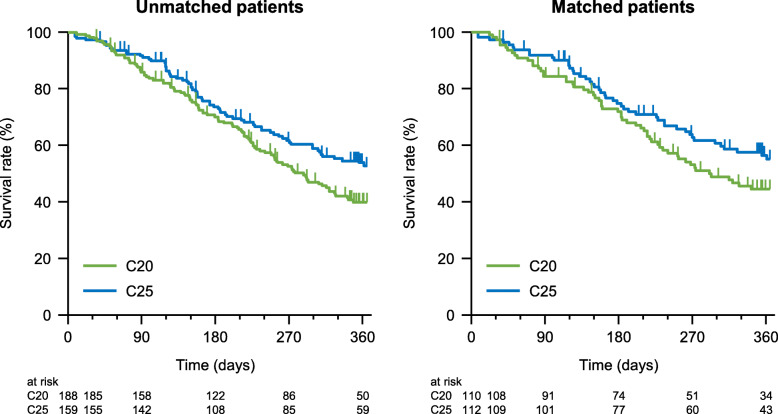
Kaplan–Meier plots of overall survival in unmatched and matched patients. C20, 20 mg/m^2^ cabazitaxel; C25, 25 mg/m^2^ cabazitaxel

The HR for OS favored C25 in the unadjusted analysis (HR 0.71, 95% CI 0.53–0.97, *p* < 0.05) and in the multivariable analysis (HR 0.69, 95% CI 0.48–0.99, *p* < 0.05). Median OS was 319 days (95% CI 293–361 days) in the overall cohort of 656 patients with available data (Table 4). Kaplan–Meier plots of OS are shown in Fig. 2 for the unmatched and matched groups. In unmatched patients, the median OS was 287 days (95% CI 240–326) in the C20 group and was not reached in the C25 group. In matched patients, the median OS was 291 days (95% CI 230–not reached) in the C20 group and was not reached in the C25 group. After applying PSM, the HR for the comparison of OS was 0.73 (95% CI 0.50–1.08), suggesting a tendency to favor C25 (Table 4).

#### TTF

**Fig. 3:**
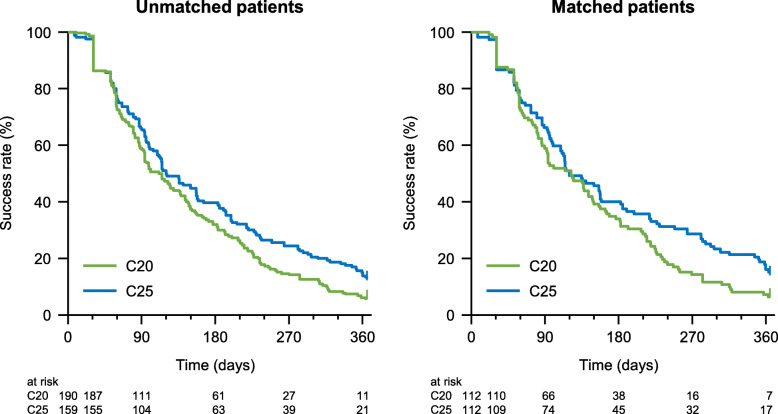
Kaplan–Meier plots of time-to-treatment failure in unmatched and matched patients. C20, 20 mg/m^2^ cabazitaxel; C25, 25 mg/m^2^ cabazitaxel

TTF was assessed in all 660 patients, including 190 in the C20 group and 159 in the C25 group. As indicated in Table 4 and Fig. 3, although the median TTF was fairly similar in the C20 and C25 groups, without and with matching, the treatment discontinuation rate increased more rapidly in the C20 group with a steeper Kaplan–Meier curve for the second 50% of patients as compared with the C25 group. Consequently, the HR for TTF tended to favor the C25 group in unmatched (HR 0.78, 95% CI 0.62–0.97, *p* < 0.05) and matched patients (HR 0.75, 95% CI 0.57–0.99, *p* < 0.05), and in the multivariable analysis (HR 0.71, 95% CI 0.54–0.93, *p* < 0.05).

## Discussion

There are several key findings of the present analyses. Cabazitaxel exposure was greater in the C25 group in terms of cumulative dose and dose intensity, but also a longer duration of treatment and a greater number of treatment cycles (in terms of mean values), as compared with the C20 group. The frequency of ADRs was greater in the C25 group, in which neutropenia and febrile neutropenia were more frequent; the rates of other ADRs were generally consistent in both groups. The higher rate of any ADRs may reflect greater exposure to cabazitaxel and longer duration of treatment. PSA response was comparable between the C25 and C20 groups. In multivariable analysis, the cabazitaxel dose was an independent prognostic factor for both OS and TTF, which were significantly better in the C25 group. These findings were also supported by the results obtained using PSM with consistent HRs of approximately 0.7–0.8. These findings implicate that C25 has a better anti-tumor effect than C20. The more favorable TTF in the C25 group may reflect a lower discontinuation rate.

The recommended initial dose of cabazitaxel is 25 mg/m^2^ in Japan. As noted here, this dose was used as the starting dose in 159 patients (24.1%), while 190 received a starting dose of 20 mg/m^2^ (28.8%). Although the physician did not record the rationale for choosing the starting dose, we suspect a lower dose was used in consideration of safety. Indeed, the rate of any ADRs was lower in the C20 group than in the C25 group (78.4% vs 89.3%). This difference was driven by differences in the rates of neutropenia and febrile neutropenia. There were no apparent differences in the rates of other ADRs between the two doses. TTF favored the C25 group, suggesting that fewer patients in this group discontinued treatment due to ADRs.

The safety of these two doses was also compared in an international, randomized controlled trial (PROSELICA), in which 598 patients were randomized to C20 and 602 to C25 [16]. Similar to the present study, treatment-emergent AEs were more frequent in the C25 group (any grade and grade ≥ 3: 93.9 and 54.5%) than in the C20 group (91.2 and 39.7%). In that study, diarrhea, neutropenia, leukopenia, and thrombocytopenia were more frequent in the C25 group.

In terms of efficacy, the PSA response rate was significantly greater in the C25 group in PROSELICA (42.9% vs 29.5%, *p* < 0.001). In our study, the PSA response rate was similar in both groups (~ 30% depending on the analysis and matching), although we defined the PSA response as a ≥30% decrease in patients with a baseline ≥5 ng/mL. Results of the present study also suggest that OS and TTF were more favorable in the C25 group than in the C20 group. In PROSELICA, the median OS was 13.4 months in the C20 group (95% CI 12.19–14.88 months), which was non-inferior to the C25 group with a median of 14.5 months (95% CI 13.47–15.28 months; HR 1.024). C25 was associated with a slightly longer progression-free survival (3.5 vs 2.9 months) and greater PSA response (42.9% vs 29.5%, *p* < 0.001) but not in terms of other efficacy endpoints [16]. The RDI in that study was almost 100% in both groups. However, in the present study, the median RDIs were 65.9 and 77.0% in the C20 and C25 groups, equivalent to doses of approximately 16 mg and 20 mg, respectively. Some discrepancies in the results may be expected considering the different patient backgrounds of the two studies (highly selected in PROSELICA vs heterogeneous in our study) and potentially differences in the management of toxicities in clinical practice. It is important to confirm the results of clinical trials in clinical practice because there are often differences in outcomes between clinical trials and clinical practice, known as the efficacy–effectiveness gap, in the field of oncology [19].

Subgroup analyses of the PROSELICA study also revealed that OS favored C25 in patients aged ≥75 years, patients with lactate dehydrogenase > 500 IU/L, and patients with treatment history of abiraterone, whereas OS favored C20 in patients with ECOG PS ≥2. In the present study, we used PSM to match patients including these factors. If these characteristics had an impact on OS in our study, we would expect the HR to become closer to 1 after matching patients by these characteristics. However, a significant difference in the HR of OS remained between the C20 and C25 groups. Therefore, the initial dose of cabazitaxel is still a confounding factor while other factors not included for the matching might have contributed to the difference in OS.

Survival might also be related to neutropenia as an index of drug exposure. Notable, post hoc analyses of the TROPIC study revealed that grade ≥ 3 neutropenia was associated with prolonged OS (16.3 vs 14.0 months, HR 0.65, *p* = 0.035) [20]. The authors speculated that this was due to insufficient drug exposure or a limited impact on the tumor-associated immune response, and they proposed continuing the intended dose of 25 mg/m^2^, if possible. In the present study, the rate of neutropenia was more common in the C25 group, and this group also showed more favorable survival. Accordingly, it is conceivable that the recommended dose was associated with a more favorable immune environment, which may have contributed to better survival in the C25 group in the multivariable analysis and in patients matched by PSM. Taken together, these findings may help justify continuing cabazitaxel at a dose of 25 mg/m^2^, although further analysis is necessary to confirm this approach.

The package insert for cabazitaxel was modified in December 2014 to allow prophylactic administration of G-CSF based on evidence showing it was effective in preventing febrile neutropenia. In our prior report [1], we confirmed that prophylactic administration of G-CSF was associated with significant reductions in the incidence of overall neutropenia events (41.1% vs 76.6%, *p* < 0.001) and febrile neutropenia (10.1% vs 16.0%, *p* = 0.032) of any grade compared with patients who did not receive G-CSF. Those results mimic those of others reported in compassionate use programs/expanded access programs in other countries in which the starting dose was 25 mg/m^2^ [21, 22]. In our prior report, we also confirmed that prophylactic G-CSF was associated with reduced frequencies of neutropenia, febrile neutropenia, and grade 4/5 febrile neutropenia in patients divided by the starting dose of cabazitaxel (15 to < 20, 20 to < 25, or ≥ 25 mg/m^2^). Those results [17] together with the results reported by Bracarda et al. [21] and Malik et al. [22] demonstrate the importance of prophylactic G-CSF administration to reduce the risk of neutropenia and related events. It is also possible that prophylactic G-CSF may permit more patients to receive the starting dose of 25 mg/m^2^.

## Limitations

Some limitations warrant mention. First, this study was an observational study conducted in real-world settings. Therefore, the dose of cabazitaxel, duration of treatment, and patient selection were at the physician’s discretion, which may introduce some confounding. Although randomized controlled trials are most appropriate for comparisons such as ours to avoid potential confounding due to patient/disease characteristics, the use of PSM partially compensates for this possible confounding. Unfortunately, as a by-product of PSM, the number of patients in each matched group was decreased by about one-third relative to the number of available patients at each dose level, attenuating statistical power. Finally, we must acknowledge that the set observation period of up to 1 year likely contributed to the fact that median OS was not reached in the C25 group.

## Conclusions

In conclusion, the multivariable analysis and PSM analyses suggested that OS and TTF may be more favorable in the C25 group than in the C20 group. However, owing to the higher risk of ADRs in the C25 group, and considering the results of the PROSELICA and TROPIC studies, we suggest that clinicians should carefully assess the risk of clinically significant ADRs, such as neutropenia and febrile neutropenia, and the possibility of prophylactic G-CSF when selecting the starting dose of cabazitaxel for patients with CRPC. A starting dose of 20 mg/m^2^ might be appropriate in patients at high risk of clinically significant ADRs or unfit patients, whereas fit patients may be candidates for a starting dose of 25 mg/m^2^.

## Data Availability

This post-marketing surveillance was conducted under the Japanese Ministerial Ordinance on Good Post-marketing Study Practice for Drugs (GPSP), and due to the characteristics of the surveillance in the regulation, the scope of permission for data sharing is limited to the content described in the paper.

## Abbreviations

ADR: Adverse drug reaction
AE: Adverse event
C20: 20 mg/m^2^ cabazitaxel
C25: 25 mg/m^2^ cabazitaxel
CI: Confidence interval
CRPC: Castration-resistant prostate cancer
ECOG: Eastern Cooperative Oncology Group
HR: Hazard ratio
OS: Overall survival
PC: Prostate cancer
PMS: Post-marketing surveillance study
PS: Performance status
PSA: Prostate-specific antigen
PSM: Propensity score matching
TTF: Time-to-treatment failure

## References

[1] Kimura T, Egawa S (2018). Epidemiology of prostate cancer in Asian countries. Int J Urol.

[2] Allemani C, Matsuda T, Di Carlo V, Harewood R, Matz M, Niksic M (2018). Global surveillance of trends in cancer survival 2000-14 (CONCORD-3): analysis of individual records for 37 513 025 patients diagnosed with one of 18 cancers from 322 population-based registries in 71 countries. Lancet.

[3] Fujimoto N (2016). Novel agents for castration-resistant prostate cancer: early experience and beyond. Int J Urol.

[4] Mizokami A, Kadono Y, Kitagawa Y, Izumi K, Konaka H (2017). Therapies for castration-resistant prostate cancer in a new era: the indication of vintage hormonal therapy, chemotherapy and the new medicines. Int J Urol.

[5] Shiota M, Yokomizo A, Eto M (2015). Taxane chemotherapy for hormone-naive prostate cancer with its expanding role as breakthrough strategy. Front Oncol.

[6] Cornford P, Bellmunt J, Bolla M, Briers E, De Santis M, Gross T (2017). EAU-ESTRO-SIOG guidelines on prostate cancer. Part II: treatment of relapsing, metastatic, and castration-resistant prostate cancer. Eur Urol.

[7] Kakehi Y, Sugimoto M, Taoka R (2017). Evidenced-based clinical practice guideline for prostate cancer (summary: Japanese Urological Association, 2016 edition). Int J Urol.

[8] de Bono JS, Oudard S, Ozguroglu M, Hansen S, Machiels JP, Kocak I (2010). Prednisone plus cabazitaxel or mitoxantrone for metastatic castration-resistant prostate cancer progressing after docetaxel treatment: a randomised open-label trial. Lancet.

[9] Mukai H, Takahashi S, Nozawa M, Onozawa Y, Miyazaki J, Ohno K (2014). Phase I dose-escalation and pharmacokinetic study (TED 11576) of cabazitaxel in Japanese patients with castration-resistant prostate cancer. Cancer Chemother Pharmacol.

[10] Nozawa M, Mukai H, Takahashi S, Uemura H, Kosaka T, Onozawa Y (2015). Japanese phase I study of cabazitaxel in metastatic castration-resistant prostate cancer. Int J Clin Oncol.

[11] Bouchard H, Semiond D, Risse M, Vrignaud P, Fischer J, Ganellin CR, Rotella DP (2012). Novel Taxanes: Cabazitaxel case study. Analogue-Based Drug Discovery III.

[12] Fumoleau P, Trigo JM, Isambert N, Semiond D, Gupta S, Campone M (2013). Phase I dose-finding study of cabazitaxel administered weekly in patients with advanced solid tumours. BMC Cancer.

[13] Mita AC, Denis LJ, Rowinsky EK, Debono JS, Goetz AD, Ochoa L (2009). Phase I and pharmacokinetic study of XRP6258 (RPR 116258A), a novel taxane, administered as a 1-hour infusion every 3 weeks in patients with advanced solid tumors. Clin Cancer Res.

[14] Pivot X, Koralewski P, Hidalgo JL, Chan A, Goncalves A, Schwartsmann G (2008). A multicenter phase II study of XRP6258 administered as a 1-h i.v. infusion every 3 weeks in taxane-resistant metastatic breast cancer patients. Ann Oncol.

[15] Pharmaceutical and Food Safety Bureau MoH, Labour and Welfare, Office of Safety I PaMDA (2015). Pharmaceuticals and Medical Devices Safety Information, No. 320.

[16] Eisenberger M, Hardy-Bessard AC, Kim CS, Geczi L, Ford D, Mourey L (2017). Phase III study comparing a reduced dose of cabazitaxel (20 mg/m(2)) and the currently approved dose (25 mg/m(2)) in postdocetaxel patients with metastatic castration-resistant prostate cancer-PROSELICA. J Clin Oncol.

[17] Suzuki K, Matsubara N, Kazama H, Seto T, Tsukube S, Matsuyama H (2019). Safety and efficacy of cabazitaxel in 660 patients with metastatic castration resistant prostate cancer in real-world settings: result of a Japanese post-marketing surveillance study. Jpn J Clin Oncol.

[18] Austin PC (2009). Balance diagnostics for comparing the distribution of baseline covariates between treatment groups in propensity-score matched samples. Stat Med.

[19] Templeton AJ, Vera-Badillo FE, Wang L, Attalla M, De Gouveia P, Leibowitz-Amit R (2013). Translating clinical trials to clinical practice: outcomes of men with metastatic castration resistant prostate cancer treated with docetaxel and prednisone in and out of clinical trials. Ann Oncol.

[20] Meisel A, von Felten S, Vogt DR, Liewen H, de Wit R, de Bono J (2016). Severe neutropenia during cabazitaxel treatment is associated with survival benefit in men with metastatic castration-resistant prostate cancer (mCRPC): a post-hoc analysis of the TROPIC phase III trial. Eur J Cancer.

[21] Bracarda S, Gernone A, Gasparro D (2014). Real-world cabazitaxel safety: the Italian early-access program in metastatic castration-resistant prostate cancer. Future Oncol.

[22] Malik Z, Di Lorenzo G, Pichler A (2020). Effect of baseline characteristics on cabazitaxel treatment duration in patients with metastatic castration-resistant prostate cancer: a post hoc analysis of the compassionate use/expanded access programs and CAPRISTANA registry. Cancers (Basel).

